# A casein hydrolysate based formulation attenuates obesity and associated non-alcoholic fatty liver disease and atherosclerosis in LDLr-/-.Leiden mice

**DOI:** 10.1371/journal.pone.0180648

**Published:** 2017-07-05

**Authors:** Marieke H. Schoemaker, Robert Kleemann, Martine C. Morrison, Joanne Verheij, Kanita Salic, Eric A. F. van Tol, Teake Kooistra, Peter Y. Wielinga

**Affiliations:** 1 Mead Johnson Pediatric Nutrition Institute, Global R&D, Nijmegen, the Netherlands; 2 TNO, the Netherlands Organization for Scientific Research, Metabolic Health Research, Leiden, the Netherlands; 3 Department of Pathology, Academic Medical Center, University of Amsterdam, Amsterdam, the Netherlands; University of Basque Country, SPAIN

## Abstract

**Background:**

Obesity frequently associates with the development of non-alcoholic fatty liver disease (NAFLD) and atherosclerosis. Chronic inflammation in white adipose tissue (WAT) seems to be an important driver of these manifestations.

**Objective:**

This study investigated a combination of an extensively hydrolyzed casein (eHC), docosahexaenoic acid (DHA), arachidonic acid (ARA), and Lactobacillus Rhamnosus *GG* (LGG) (together referred to as nutritional ingredients, NI) on the development of obesity, metabolic risk factors, WAT inflammation, NAFLD and atherosclerosis in high-fat diet-fed LDLr-/-.Leiden mice, a model that mimics disease development in humans.

**Methods:**

LDLr-/-.Leiden male mice (n = 15/group) received a high-fat diet (HFD, 45 Kcal%) for 21 weeks with or without the NI (23.7% eHC, 0.083% DHA, 0.166% ARA; all w/w and 1x10^9^ CFU LGG gavage 3 times/week). HFD and HFD+NI diets were isocaloric. A low fat diet (LFD, 10 Kcal%) was used for reference. Body weight, food intake and metabolic risk factors were assessed over time. At week 21, tissues were analyzed for WAT inflammation (crown-like structures), NAFLD and atherosclerosis. Effects of the individual NI components were explored in a follow-up experiment (n = 7/group).

**Results:**

When compared to HFD control, treatment with the NI strongly reduced body weight to levels of the LFD group, and significantly lowered (P<0.01) plasma insulin, cholesterol, triglycerides, leptin and serum amyloid A (P<0.01). NI also reduced WAT mass and inflammation. Strikingly, NI treatment significantly reduced macrovesicular steatosis, lobular inflammation and liver collagen (P<0.05), and attenuated atherosclerosis development (P<0.01). Of the individual components, the effects of eHC were most pronounced but could not explain the entire effects of the NI formulation.

**Conclusions:**

A combination of eHC, ARA, DHA and LGG attenuates obesity and associated cardiometabolic diseases (NAFLD, atherosclerosis) in LDLr-/-.Leiden mice. The observed reduction of inflammation in adipose tissue and in the liver provides a rationale for these comprehensive health effects.

## Introduction

The prevalence of overweight and obesity has risen dramatically in the past decades worldwide.[1] Obesity is a hallmark of the metabolic syndrome which represents a major global health problem that frequently associates with the development of non-alcohol fatty liver disease (NAFLD), and cardiovascular disease (CVD) as complications.

There are at least three critical periods for the development of obesity and its complications [2, 3]: during gestation and early infancy, in the period of adiposity rebound that occurs between 5 and 7 years of age, and in adolescence. The processes that contribute to the development of obesity have been extensively investigated and include increased calorie intake, nutrient overload, decreased physical activity, and changes in the gut microbiome.[4–7] It is thought that these processes contribute to the development of chronic inflammation in distinct adipose tissue depots, and that adipose tissue inflammation is a driving force for NAFLD and CVD development.[8–10]

Preventive nutritional strategies during critical periods in life seem promising to reduce the risk of obesity development.[11] A combination of dietary compounds that can affect multiple disease processes may be effective. We herein examined the impact of a combination of three specific nutritional ingredients (NI) on the development of obesity and its comorbidities in liver and vasculature. The specific NI are extensively hydrolyzed casein (eHC), long-chain polyunsaturated fatty acids (LC-PUFAs) Docosahexaenoic acid (DHA) and Arachidonic acid (ARA) and the probiotic strain *Lactobacillus Rhamnosus* GG (LGG). These NI are also present in infant formulas and combination treatment in early life has been shown to improve clinical health outcomes.[12–15] However, it is not known whether combination treatment with NI would counteract the detrimental effects of an obesogenic diet and prevent NAFLD and CVD on the long run.

Protein can have an impact on metabolism [16] and there are indications that casein hydrolysates exert anti-obesity effects in mice [17] and may alleviate inflammation in children.[12] Extensive casein hydrolysates are used for the dietary management of cow milk allergy, and hence are devoid of allergenic protein isotopes. But despite lacking proteins, these eCH contain smaller functional peptide sequences that may have a variety of biological effects, which are poorly understood. Extensive enzyme hydrolysis generates bioactive sequences and predominantly produces smaller peptides (95% lower than 1 kDa). As such, extensive casein hydrolysates can vary in peptide moieties and in milk proteins with unique bioactive sequences.[18] Because of the unique properties of each hydrolysate, general health claims should not be made and dedicated studies are therefore required to better describe the functional peptide composition of these eCH in relation to their broader biological activity. In the case of LC-PUFAs, it has been proposed that their intake lowers adiposity and attenuates related complications, both in pre-clinical and clinical studies. However, most of these studies investigated the effects of the n-3 LC-PUFA DHA [19–21], whereas supplementation with n-6/n-3 LC-PUFAs at a particular ratio (2:1) has been associated with health effects in humans and animal studies.[14, 15, 22, 23] We therefore tested a well-defined n-6/n-3 LCPUFA mixture of ARA/DHA as present in breast milk. In addition to eHC and LC-PUFAs, we also employed a well characterized probiotic LGG that has been shown to affect inflammatory responses in children [24] and that may ameliorate experimental NAFLD.[25]

The aim of the present study is to investigate whether this combination of NI (eHC, ARA/DHA, LGG) may counteract the development of obesity and associated NAFLD and atherosclerosis. To do so, we used LDLr-/-.Leiden mice, which develop obesity and risk factors of the metabolic syndrome, and pathological endpoints akin to humans.

## Methods

### Animals and diets

Experiments were performed conform to the rules and regulations set forward by the Netherlands Law on Animal Experiments and were approved by an independent Committee on Animal Care and Experimentation (Dierexperimentencommissie Zeist, Netherlands; approval number 3277). Male low-density lipoprotein receptor-deficient LDLr-/-.Leiden mice were obtained from the breeding facility at TNO. Animals were housed in macrolon cages (3–5 mice per cage) during the experiment in clean-conventional animal rooms (relative humidity 50–60%, temperature ~21°C, light cycle 7 am to 7 pm). Food and acidified tap water were supplied *ad lib*. Mice were fed standard lab chow (Ssniff R/M diet V1530, Uden, The Netherlands) until the start of the study at 12–15 weeks of age (that is when mice were young adolescent). Mice were divided (at t = 0) into three experimental groups (*n* = 15 per group) that were matched based on blood glucose (primary matching parameter) and body weight (secondary matching parameter). Animals were fed for 21 weeks a high fat diet (HFD, 45 Kcal%) with or without specific NI or a low fat diet (LFD, 10%Kcal) as a reference. The LFD (diet D12450B, Research Diets, New Brunswick, USA) contained 19.2% w/w protein, 67.3% w/w carbohydrates and 4.3% w/w fat. The high fat diet (HFD) control group was fed a lard based diet (diet D12451 Research Diets, New Brunswick, USA) containing 23.7% w/w protein, 41.4% w/w carbohydrates and 23.6% w/w fat. Control mice were treated three times a week with PBS gavage (200 μL) to control for the effect of gavage treatment in the experimental group treated with NI. This group was fed the same high fat diet as the HFD control group but the intact casein protein was fully replaced by extensively hydrolyzed casein (HFD+NI diet, MJN, Evansville, IN), and the diet was supplemented with 0.083% w/w DHA and 0.166% w/w ARA (both from DSM Nutritional Products North America, Columbia, MD, USA). Three times per week (Monday, Wednesday, Friday at 11 a.m.), mice received *Lactobacillus Rhamnosus GG* (LGG, Chr. Hansen Holding A/S. Denmark) at 1x10^9^ CFU in 200 μL of PBS through oral gavage. The composition of the diets of the three groups is specified in S1 Table.

Additionally, an explorative follow-up study was performed (n = 7/group) under the same experimental conditions as described above, i.e. groups of mice were treated with HFD and a single component of the NI as well as the NI mixture for 21 weeks to explore the effects of the single components on body weight, metabolic risk factors and liver pathology.

### Body composition and food intake

Body weight (individually) and food intake (at cage level, n = 3–5 mice per cage) were monitored over time. Food intake was analyzed in week 3, 6, 9, 12, 15, 18 and 21 on the diets. In each week, food intake was assessed over a period of five days and then the average daily food intake in gram per mouse was calculated.

After 21 weeks on the diets, total body fat and lean body mass were assessed non-invasively by EchoMRI (EchoMRI LLC, Houston TX, USA). Conscious mice were placed in a constraint tube which was inserted into the EchoMRI for a period of approximately 30 s. During that time, total body fat and lean body mass were measured.

### Plasma and urine measurements

Blood samples were taken by tail incision in week 0, 3, 6, 9, 12, 15, 18 and 21 after 5 h fasting allowing a collection of approximately 50 μl plasma. Blood glucose was measured immediately using a hand-held glucometer in tail blood (FreeStyle Lite, Abbott, Alameda CA, USA). The remainder of the plasma was used for plasma lipid analysis, or stored at -80°C for further analysis. Total plasma cholesterol and triglyceride levels were measured using kits No. 11489437 and 11488872 (Roche Diagnostics, Almere, The Netherlands), respectively. Plasma alanine aminotransferase (ALAT) levels were measured using a spectrophotometric activity assay (Reflotron-Plus, Roche). Fasting plasma insulin (Ultrasensitive mouse insulin ELISA, Mercodia, Uppsala, Sweden), soluble vascular cell adhesion molecule 1 (sVCAM-1; R&D Systems), leptin (R&D Systems), adiponectin (R&D Systems) and serum amyloid A (SAA; Biosource) were determined by ELISA in EDTA plasma. Serum C-peptide, resistin and GIP were determined by the ‘Millipore metabolic hormones Multiplex kit’ (MMHMAG-44K). The beads were read on a LiquiChip 200, (Qiagen, Hombrechtikon, Switzerland), and data were analyzed by the five parameter curve fitting in Luminex100 IS Software. HOMA-index was calculated as previously described.[26] To assess glomerular function, urine was collected during the study and urinary albumin (Exocell Inc. Philadelphia, PA, USA) and creatinine concentrations were determined (Bethyl Laboratories Inc. Montgomery, TX, USA) according to the instructions of the manufacturers.

### Sacrifice

Animals were not fasted at sacrifice. The mice were sacrificed after 21 weeks between 10 a.m. and 2 p.m. and liver and adipose tissue from inguinal, omental and epididymal depots were isolated for further analyses. Blood was collected by heart puncture to prepare serum. The heart including aortic root was used for atherosclerosis analysis.

### Adipose tissue analysis

From all three adipose tissue depots (inguinal, omental and epididymal), cross-sections were prepared from paraffin-embedded samples and stained with hematoxylin-phloxine-saffron. From each mouse and each fat depot, three cross-sections were evaluated for the presence of crown-like structures. The analyzed surface area of each cross-section was 580.000 μm^2^, resulting in an analyzed area of in total 1.74 mm^2^.

### Liver tissue analyses

Formalin-fixed and paraffin-embedded cross-sections (5μm) of the median lobe were stained with haematoxylin and eosin and scored blindly by a board-certified pathologist using an adapted grading method for human NASH [27]. Briefly, two HE-stained cross-sections/mouse were examined and the level of macrovesicular steatosis was determined relative to the liver area analyzed (expressed as a percentage). In addition, liver lipids were analyzed biochemically by high-performance thin-layer chromatography (HPTLC) as described previously.[28] Briefly, lipids were extracted from freshly prepared liver homogenates following the Bligh and Dyer method [29] after which they were separated by HPTLC on silica gel plates. Then, lipid spots were stained with color reagent (5 g of MnCl_24_H_2_O, 32 ml of 95–97% H_2_SO_4_ added to 960 ml of CH_3_OH/H_2_O = 1:1 (v/v)). Hepatic triglycerides, cholesteryl esters and free cholesterol were quantified using Image Lab, version 5.2.1 (Bio-Rad Laboratories, Veenendaal, the Netherlands) and expressed per mg liver protein, which was measured in the same homogenates used for the HPTLC analysis using the Lowry Protein assay.[30]

Hepatic inflammation was assessed by counting the number of inflammatory foci per field at a 100× magnification (view size 3.1 mm^2^) in five non-overlapping fields per HE-stained specimen, expressed as the average number of foci per field. Fibrosis was assessed histochemically by Picro-Sirius Red staining (Chroma, WALDECK-Gmbh, Münster, Germany) and visualized using ImageJ software (version 1.48, NIH, Bethesda, MD, USA). The hepatic collagen content was quantified biochemically in freshly prepared homogenates of liver tissue using a total collagen assay (Quickzyme, Leiden, The Netherlands). This assay does not discriminate between different collagen types, and reflects the total hepatic collagen content. To further investigate effects on inflammation and fibrosis, hepatic gene expression was assessed by RT-qPCR. For this, RNA was extracted from snap-frozen liver tissue using RNA-Bee Total-RNA Isolation Kit (Bio-Connect, Huissen, the Netherlands). Nanodrop 1000 (Isogen Life Science, De Meern, the Netherlands) was used for spectrophotometric assessment of RNA concentration and RNA integrity was evaluated using 2100 Bioanalyzer (Agilent Technologies, Amstelveen, the Netherlands). cDNA was synthesized from 1 μg of RNA using a High-Capacity RNA-to-cDNA Kit (Life Technologies, Bleiswijk, the Netherlands). Transcripts were quantified using TaqMan Gene Expression Assays (Life Technologies) and the following primer/probe-sets: *Tnf* (Mm00443258_m1), *Cd68* (Mm03047340_m1), *Ccl2* (Mm00441242_m1), *Tgfb1* (Mm00441724_m1), *Col1a1* (Mm00801666_g1), and *Col5a1* (Mm00489342_m1). *Ppif* (Mm01273726_m1) and *Hprt* (Mm00446968_m1) were used as endogenous controls. Changes in gene expression were calculated using the comparative Ct (ΔΔCt) method and expressed as fold-change relative to LFD as described previously.[31]

### Atherosclerosis analysis

Atherosclerosis was analyzed blindly in hematoxylin-phloxinesaffron-stained serial cross-sections (n = 4 of each mouse) of the aortic arch (40 mm intervals) and scored essentially as described [32] using an Olympus BX51 microscope and CelîD software (Olympus, Zoeterwoude, The Netherlands).

### Fecal energy content

Fecal samples at cage level (n = 3–5 mice per cage) were collected over a fixed period of one week in week 1, week 9 and week 21 of dietary exposure. Samples were dried and homogenized and energy content was assessed as a measure of reduced intestinal energy uptake. This was performed by using bomb calorimetry (IKA oxygen bomb calorimetry, company, country). Data were expressed as kJ/g feces and compared to food intake and body weight.

### Statistical analysis

SPSS Version 20 was used for statistical evaluation of the data. Two-Way repeated measures ANOVA with factors time and diet were applied to analyze for a diet, time and interaction effect. One-Way ANOVA for individual time points were applied to analyze for differences at specific time points between groups. LSD post hoc test was used to compare groups. For non-parametric comparison, Mann-Whitney U tests were performed. One mouse in the LFD and one mouse in the experimental diet group were excluded from the data set and all analyses because they were statistical and biological outliers. P<0.05 was considered significant. All data are presented as mean ± SEM.

## Results

### Reduced body weight gain at higher food intake in mice fed HFD+NI

**Fig 1:**
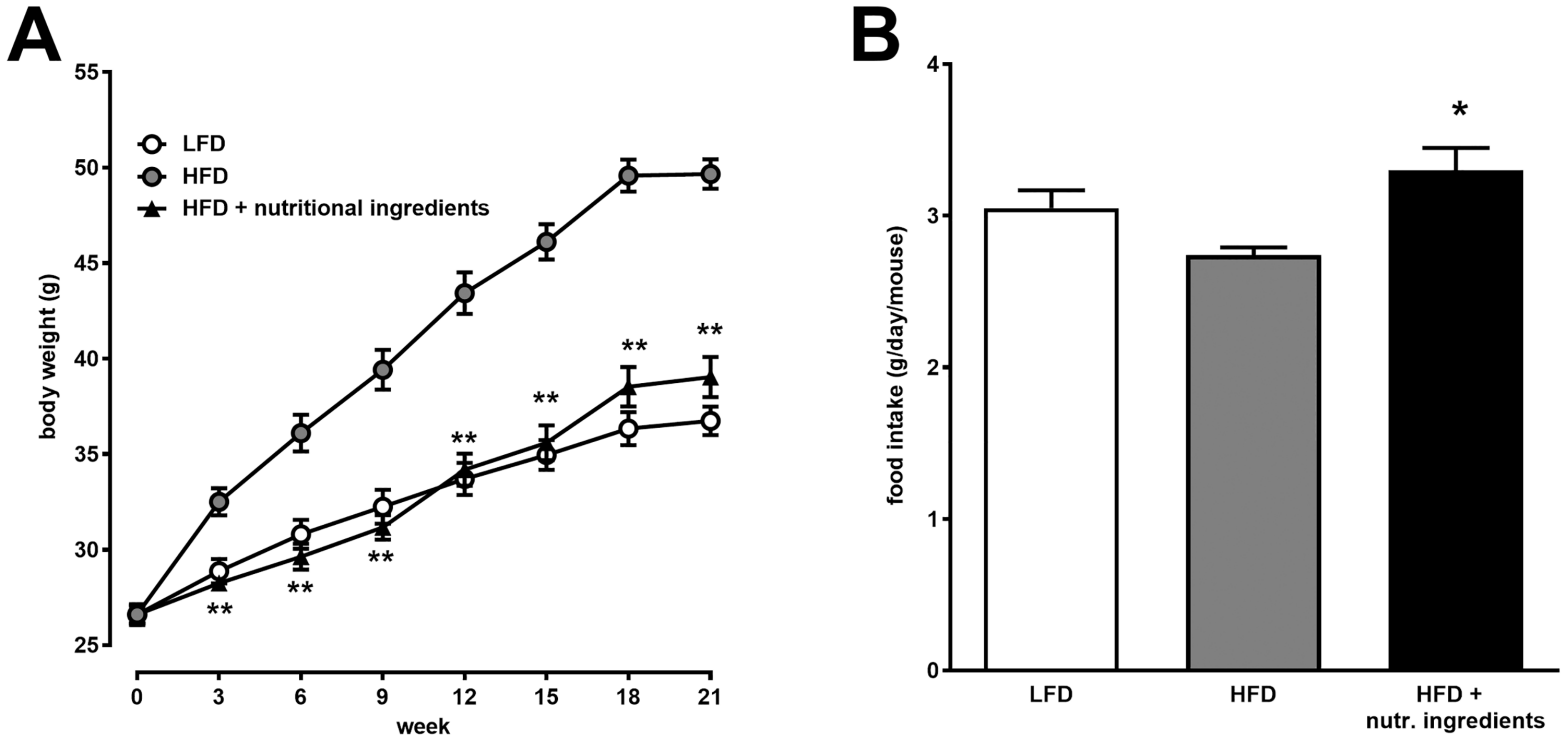
Effects of nutritional ingredients on body composition and food intake in LDLr-/-.Leiden mice. Changes in Body weight over time (A) and average daily food intake (B). Data are presented as mean ± SEM, n = 15. Significant diet effects are shown by *P<0.05 or **P<0.01 compared to HFD. LFD = Low Fat Diet; HFD = High Fat Diet + control gavage; HFD + nutritional ingredients (including eHC, ARA, DHA, and gavage with LGG).

The dietary interventions with HFD or HFD+NI started when LDLr-/-.Leiden mice were young adolescent (12 weeks of age). Twenty-one weeks of HFD feeding with PBS gavage control resulted in a body weight gain of 25 grams in the HFD control group while LFD fed mice gained approximately 10 grams (Fig 1A). Body weight and body weight gain of mice fed HFD+NI were significantly lower than HFD fed mice and comparable to LFD fed mice.

Food intake in the HFD+NI group was significantly higher compared to HFD (Fig 1B). There was no effect on the fecal energy content which was determined as a measure of intestinal energy uptake (data not shown). Together these data indicate that animals fed HFD+NI can maintain a low body weight under obesogenic conditions, despite higher food intake.

### Intervention with NI reduces metabolic and cardiovascular risk factors

**Fig 2:**
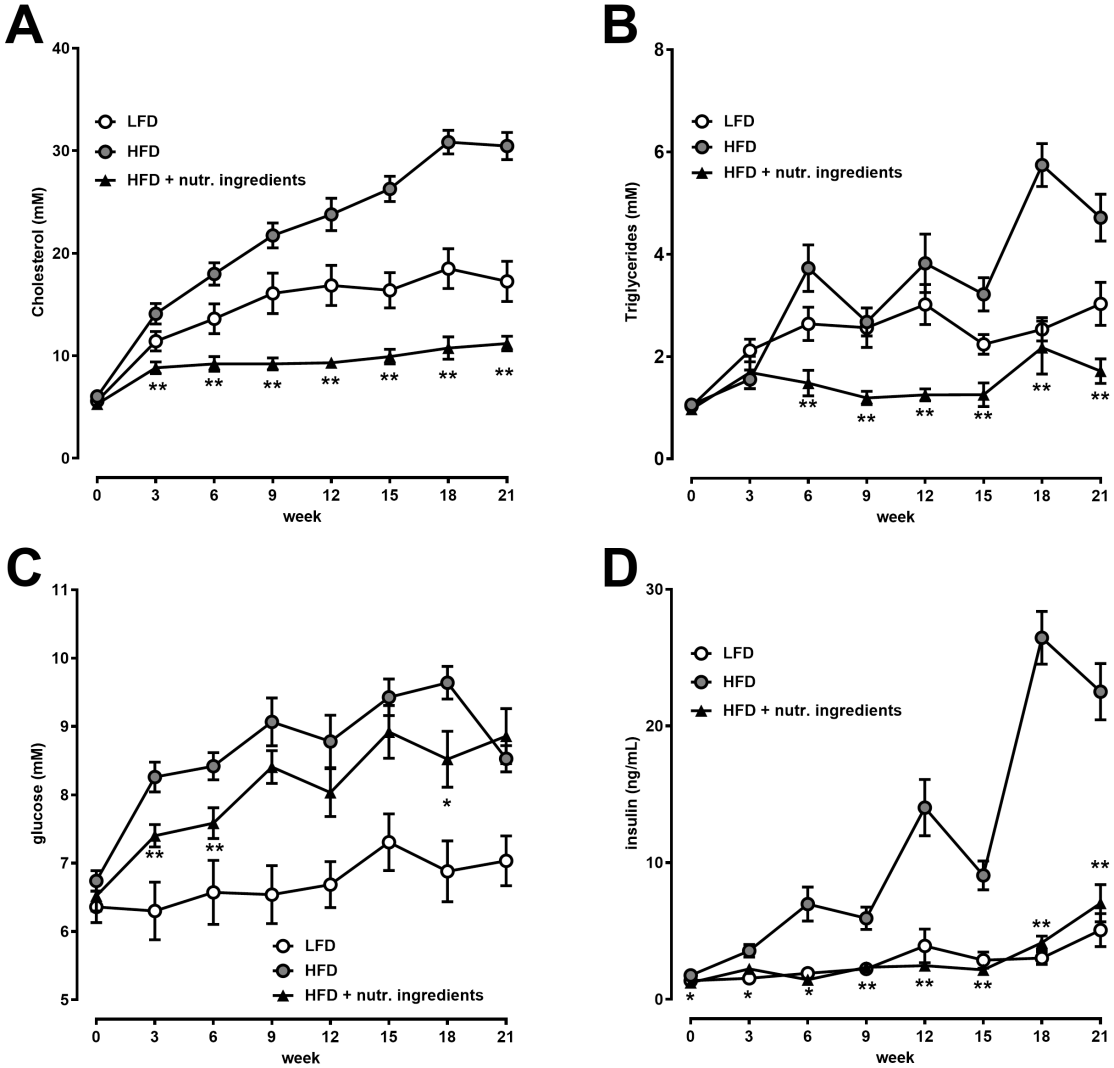
Effects of nutritional ingredients on cardiovascular risk factors in LDLr-/-.Leiden mice. Plasma levels at 21 weeks of dietary intervention for cholesterol (A), triglycerides (B), glucose (C), insulin (D). Values are presented as mean ± SEM, n = 15. Significant diet effects are shown by *P<0.05 or **P<0.01 compared to HFD. LFD = Low Fat Diet; HFD = High Fat Diet + control gavage; HFD + nutritional ingredients (including eHC, ARA, DHA, and gavage with LGG).

Plasma cholesterol increased gradually over time in the HFD group whereas plasma cholesterol in the LFD group increased modestly until week 9 and remained stable for the remainder of the study (Fig 2A). The HFD+NI group had low levels of plasma cholesterol throughout the study until 21 weeks (Fig 2A). Similarly, plasma triglycerides remained low in the HFD+NI group (Fig 2B) which underscored the pronounced effects on plasma lipids. Fasting blood glucose levels increased in the HFD group while they remained low in the LFD group. At several time points, blood glucose in the HFD+NI group was significantly lower than in the HFD group (Fig 2C). Plasma fasting insulin levels gradually increased over time in the HFD group whereas they remained low in the HFD+NI group comparable to the LFD group showing a significant difference versus HFD throughout the entire experiment (Fig 2D). In line with this, C-peptide, which is co-secreted with insulin, was significantly lower in the HFD+NI group compared to the HFD control group (Table 1). Also the HOMA-index data indicated that NI significantly reduced insulin resistance relative to HFD (Table 1).

**Table 1:** Effects of nutritional ingredients on metabolic hormones in LDLr-/-.Leiden mice.

	LFD	HFD	HFD + NI
**C-Peptide (ng/mL)**	6.7 ± 1.6	38.2 ± 5.2	10.1 ± 2.0**
**GIP (pg/mL)**	151 ± 35	194 ± 18	142 ± 11*
**Resistin (ng/mL)**	9.7 ± 1.1	22.9 ± 1.8	20.1 ± 1.6
**Leptin (ng/mL)**	12.6 ± 2.0	52.5 ± 2.7	20.6 ± 2.5**
**Adiponectin (μg/mL)**	6.9 ± 2.6	4.4 ± 1.0	5.3 ± 1.3^$^
**HOMA-index**	1.64 ± 1.54	8.65 ± 3.54	3.0 ± 2.59***

LFD = Low Fat Diet; HFD = High Fat Diet + control gavage; HFD + nutritional ingredients (NI, including eHC, ARA, DHA, and gavage with LGG). Significant diet effects are shown by

*P<0.05 or

**P<0.01 or

***P<0.001 or

^$^P = 0.07 compared to HFD.

**Fig 3:**
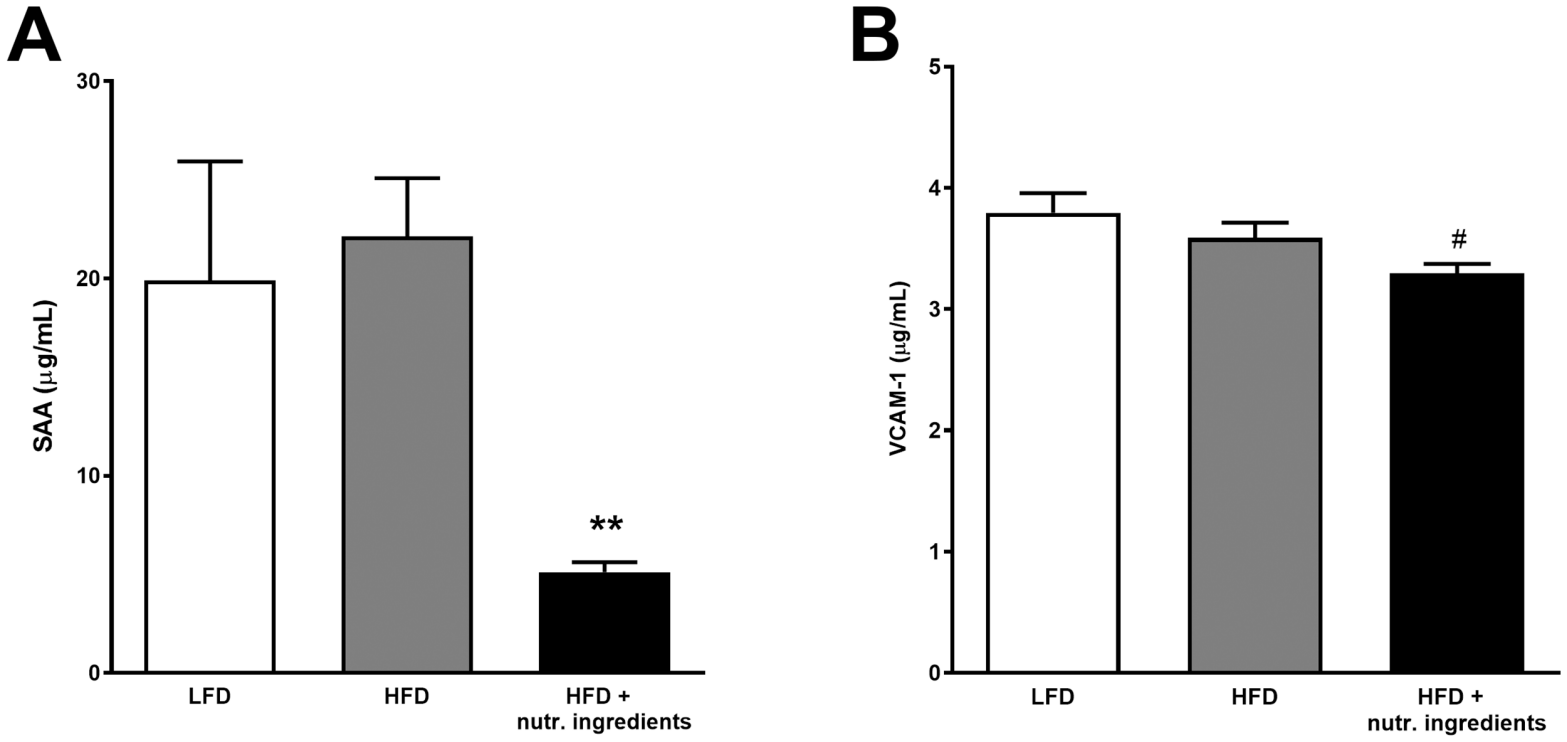
Effects of nutritional ingredients on markers of systemic inflammation and vascular activation in LDLr-/-.Leiden mice. Plasma levels of SAA (A) and VCAM-1 (B) at 21 weeks of dietary exposure. Data are presented as mean ± SEM, n = 15 Significant diet effects are shown by **P<0.01 or ^#^P = 0.06 compared to HFD. LFD = Low Fat Diet; HFD = High Fat Diet + control gavage; HFD + nutritional ingredients (including eHC, ARA, DHA, and gavage with LGG).

Serum Amyloid A (SAA), a marker of systemic inflammation, and VCAM-1, a marker for vascular activation were comparable in LFD and HFD. SAA was significantly lower in the HFD+NI group compared to HFD (Fig 3A) and VCAM-1 tended to be lower (Fig 3B). Additionally metabolic hormones GIP and leptin were significantly lower in the HFD+NI group (Table 1). Overall, NI did not correct HFD-induced metabolic parameters to the level of LFD but typically attenuated the effect of HFD. Adiponectin plasma levels were higher in the HFD+NI group when compared to HFD with borderline significance (P = 0.07). No significant effects were observed for resistin plasma levels (Table 1).

### NI attenuates HFD-induced adiposity and adipose inflammation

**Fig 4:**
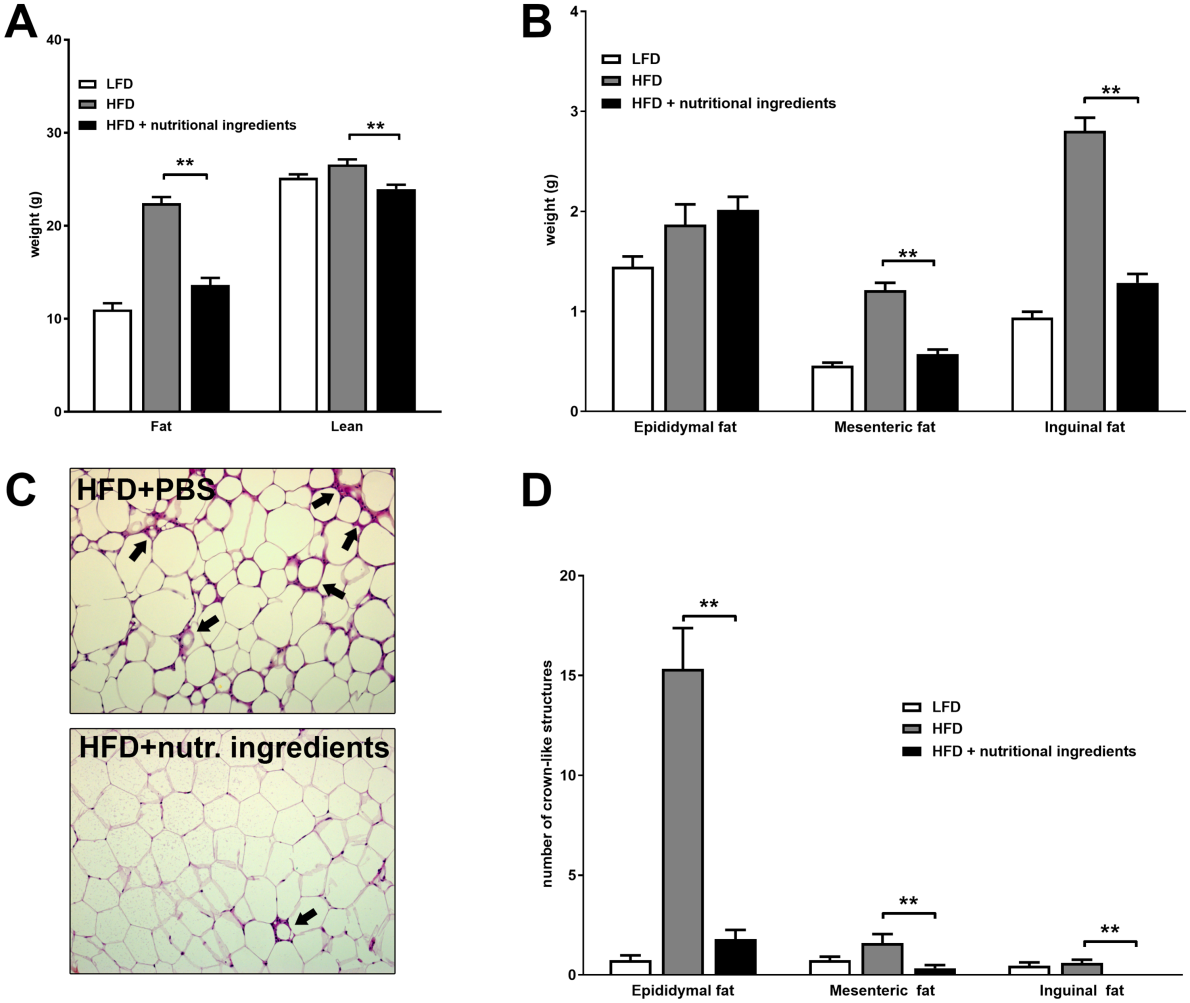
Effects of nutritional ingredients on adipose tissue quantity and quality in LDLr-/-.Leiden mice. Body composition analysis at 21 weeks of feeding regimes with fat and lean mass (A), with weights of epididymal fat, mesenteric fat and inguinal fat depots (B), representative histology of epidydimal WAT with inflammatory cells forming crown like structures (CLS, black arrows) (C) and quantitative analysis of CLS in different adipose tissue depots (D) (C). Data are presented as mean ± SEM, n = 15. Significant diet effects are shown by **P<0.01 compared to HFD. LFD = Low Fat Diet; HFD = High Fat Diet + control gavage; HFD + nutritional ingredients (including eHC, ARA, DHA, and gavage with LGG).

Total body composition after 21 weeks of dietary intervention was analyzed non-invasively by EchoMRI (Fig 4A). Mice in the HFD+NI group had 39% less body fat and 10% less lean body mass compared with mice fed a HFD, showing a body composition similar to LFD fed mice. After sacrifice, adipose tissues were isolated and weighed to investigate the impact of dietary regimens on fat distribution between these depots. The epididymal fat depot was comparable between the HFD+NI group and the HFD group, while tissue weights from the mesenteric and inguinal fat depots were strongly reduced by the nutritional ingredients (reduction of 52% and 54% respectively) and more closely matching adipose tissues from LFD fed mice (Fig 4B). Additionally, the number of crown-like structures were counted as a measure of inflammatory cell infiltration in the adipose tissue depots (Fig 4D and representative photomicrographs in Fig 4C). The HFD group developed a high number of crown like structures in the epididymal adipose tissue, while mice fed HFD+NI showed hardly any crown-like structures in this depot. Although in the mesenteric and inguinal depots only few crown-like structures were induced by HFD, still these were significantly lowered and hardly present in the HFD+NI group.

### NI protects against development of hepatosteatosis and liver inflammation

**Fig 5:**
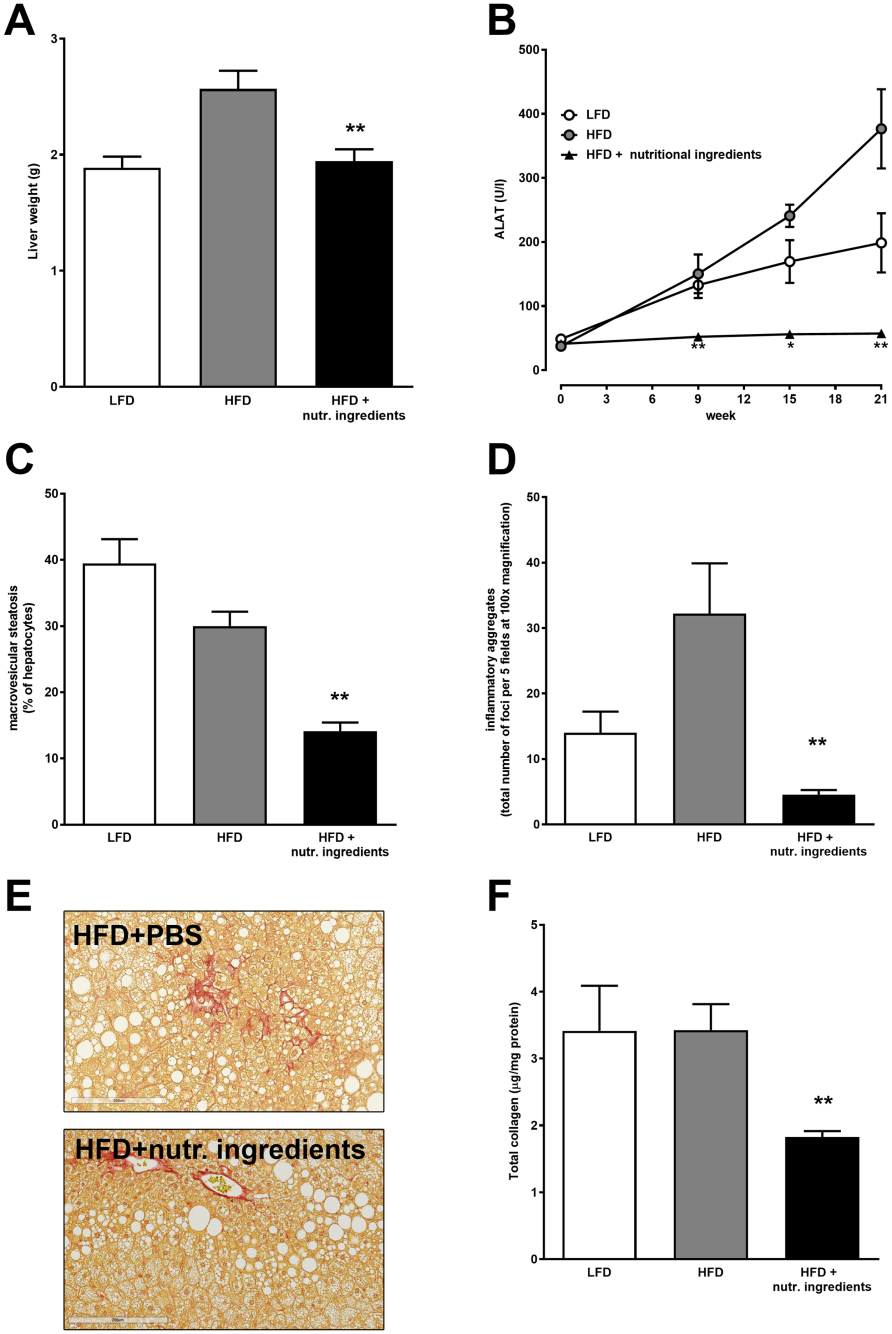
Liver integrity and non-alcoholic fatty liver disease in LDLr-/-.Leiden mice. Liver mass at 21 weeks of feeding regimes (A), alanine aminotransferase (ALAT) over time (B), liver macrovascular steatosis (C) and lobular inflammation (D). Representative Sirius Red staining of liver (E) and quantitative biochemical analysis of hepatic total collagen (F) at 21 weeks of dietary interventions. Data are presented as mean ± SEM, n = 15. Significant diet effects are shown by *P<0.05 or **P<0.01. Low Fat Diet; HFD = High Fat Diet + control gavage; HFD + nutritional ingredients (including eHC, ARA, DHA, and gavage with LGG).

Liver integrity was assessed by analysis of circulating ALAT levels. HFD mice showed a substantial increase in ALAT levels over time, indicating liver damage. ALAT levels of the HFD+NI group remained low throughout the experiment and were significantly lower compared to HFD control at all time points (Fig 5B). The liver weight of the HFD control group was markedly higher, while liver weight in the HFD+NI group was significantly lower and comparable to LFD reference animals (Fig 5A). Macrovesicular steatosis and lobular inflammation, both hall marks of non-alcoholic fatty liver disease were markedly and significantly lower in HFD+NI fed mice compared to HFD (Fig 5C and 5D respectively). Also the formation of total collagen (an indicator of fibrosis) was significantly attenuated in the HFD+NI group (Fig 5E and 5F).

These anti-steatotic, anti-inflammatory and anti-fibrotic effects were confirmed with independent biochemical measurement of hepatic lipids (triglycerides, cholesteryl esters and free cholesterol) and gene expression analysis of inflammation and fibrosis-related genes (i.e.. TNFα, CD68, MCP1 and TGFβ, Col1A1, Col5A1) (Table 2). These analyses showed that the anti-steatotic effects of NI were attributable to significant reductions in hepatic triglyceride levels as well as reduced levels of both unesterified (free) cholesterol and esterified cholesterol (Table 2). Hepatic gene expression analyses showed that NI attenuated expression of the pro-inflammatory cytokine TNFα and the macrophage marker CD68, while expression of the chemokine MCP-1 was not affected by NI. Addition of NI to the diet significantly reduced expression of the pro-fibrotic cytokine TGFβ and alpha-1 type I collagen. Expression of alpha-1 type V collagen was not affected.

**Table 2:** Effects of nutritional ingredients on hepatic lipids and expression of hepatic genes.

	LFD	HFD	HFD + NI
**Liver lipids (μg/mg protein)**			
liver triglycerides	181.8 ± 23.2**	227.7 ± 18.2	176.0 ± 25.3**
liver cholesteryl ester	21.9 ± 3.1*	27.4 ± 6.1	14.7 ± 3.4**
liver free cholesterol	16.8 ± 1.9	17.5 ± 1.8	15.1 ± 0.8*
**Hepatic gene expression (fold change relative to LFD)**			
tumor necrosis factor alpha (*Tnf)*	1.00 ± 0.23	1.32 ± 0.53	0.61 ± 0.17**
CD68 (*Cd68*)	1.00 ± 0.20	1.10 ± 0.25	0.78 ± 0.53**
monocyte chemoattractant protein 1 (*Ccl2*)	1.00 ± 0.42	0.84 ± 0.74	1.55 ± 1.22
TGF-β (*Tgfb1*)	1.00 ± 0.20	1.30 ± 0.42	0.89 ± 0.20*
alpha-1 type I collagen (*Col1a1*)	1.00 ± 0.12**	3.45 ± 2.92	0.56 ± 0.13***
alpha-1 type V collagen (*Col5a1*)	1.00 ± 0.18	1.15 ± 0.80	0.89 ± 0.20

LFD = Low Fat Diet; HFD = High Fat Diet + control gavage; HFD + nutritional ingredients (NI, including eCH, ARA, DHA, and gavage with LGG). Significant diet effects are shown by

*P<0.05,

**P<0.01,

***P<0.001 compared to HFD.

### NI exerts anti-atherosclerotic vasculoprotective effects

**Fig 6:**
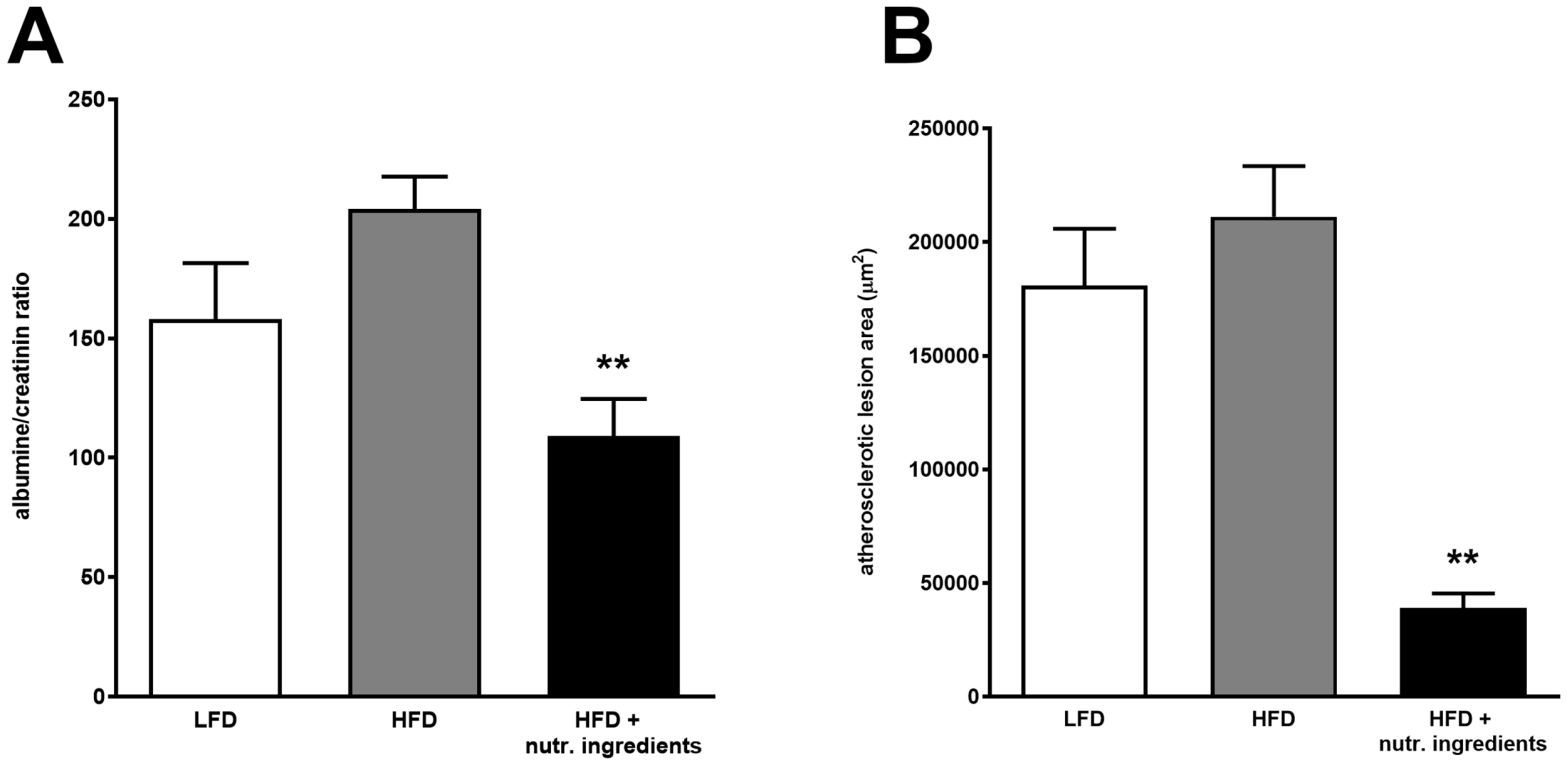
Vasculoprotective effects of nutritional ingredients in LDLr-/-.Leiden mice. Albuminurea at 15 weeks (A) and Atherosclerosis (B) at 21 weeks of dietary interventions. Data are presented as mean ± SEM, n = 15. Significant diet effects are shown by *P<0.05 or **P<0.01 compared to HFD. Low Fat Diet; HFD = High Fat Diet + control gavage; HFD + nutritional ingredients (including eHC, ARA, DHA, and gavage with LGG).

Analysis of the urinary albumin/creatinine ratio showed higher levels in HFD relative to LFD. Treatment with the nutritional ingredients reduced microalbuminuria significantly (Fig 6A) suggesting a beneficial effect on the microvasculature. We next analyzed atherosclerosis development in the aortic valve area and observed pronounced atherosclerosis development in both HFD and LFD treated groups with total lesion areas of 211138 μm^2^ and 180795 μm^2^, respectively. Intervention with the nutritional ingredients strongly attenuated atherosclerosis development (39083 μm^2^) further supporting a vasculoprotective effect of these specific nutritional compounds (Fig 6B and representative photomicrographs in S1 Fig).

### Health effects of NI are attributable to the combined effects of its constituents

To assess whether the observed health effects of NI can be explained by a specific constituent of NI or the combination of constituents, a small-scaled (n = 7/group) experiment was performed with its single components. As shown in Table 3, the extensively hydrolyzed casein component is largely responsible for the observed lowering effect of atherogenic plasma lipids (triglycerides and cholesterol). Other effects of the NI mixture cannot be explained by a single constituent (e.g. body weight, fat mass, insulin, ALT and liver histology). These data suggests that complimentary underlying mechanisms of action of the constituents may contribute to the health effects observed with NI.

**Table 3:** Effects of individual nutritional ingredients.

21 weeks	HFD	HFD + NI	HFD + eHC	HFD + LCPUFAs	HFD + LGG
**Body weight (g)**	48.2 ± 6.3	42.1 ± 4.8**	44.3 ± 5.0*	50.2 ± 5.4	45.8 ± 5.3
**Body composition (g)**					
Fat mass	18.4 ± 5.7	14.4 ± 3.4*	17.1 ± 3.1	21.3 ± 4.0	17.1 ± 4.6
Lean mass	28.1 ± 2.1	26.0 ± 1.7*	25.4 ± 1.8**	26.8 ± 1.3	27.1 ± 3.0
**Insulin (ng/ml)**	16.4 ± 9.1	8.2 ± 4.3^$^	12.1 ± 9.1	18.9 ± 11.9	17.6 ± 14.1
**Triglycerides (mM)**	3.5 ± 3.0	1.1 ± 0.6**	1.5 ± 1.0*	4.6 ± 3.0	3.0 ± 1.7
**Cholesterol (mM)**	24.3 ± 13.7	11.1 ± 3.3***	11.9 ± 3.1**	28.8 ± 9.9	21.4 ± 7.3
**ALT (U/L)**	187	54	67	81	124
**Liver histology**					
Macrovesicular steatosis (%)	30.3 ± 12.6	11.3 ± 6.4***	19.3 ± 5.3**	29.8 ± 10.6	23.6 ± 5.4
Inflammatory aggregates (per 5 fields)	29.4 ± 67.5	10.6 ± 6.6	7.5 ± 3.9	21.7 ± 21.5	15.6 ± 23.0

LFD = Low Fat Diet; HFD = High Fat Diet + control gavage; HFD + nutritional ingredients (NI, including eCH, ARA, DHA, and gavage with LGG). Significant diet effects are shown by

*P<0.05 or

**P<0.01 or

***P<0.001 or

^$^P = 0.07 compared to HFD (n = 7/group).

## Discussion

In this study, we investigated the combined effect of specific nutritional ingredients (NI) with beneficial health effects, i.e. an extensively hydrolyzed casein, the LC-PUFAs DHA and ARA and the probiotic LGG, on the development of obesity and its comorbidities NAFLD and atherosclerosis. The study was performed in LDLr-/-.Leiden mice which develop obesity and dyslipidemia and associated NAFLD and atherosclerosis when treated with HFD. In this translational model, the applied concentration of fat is also reached in human diets [33], and NAFLD and atherosclerosis were scored using human-based grading systems.[27, 34, 35] The tested combination of NI mitigated HFD-induced obesity and the development of associated complications, NAFLD and atherosclerosis.

The combined NI strongly attenuated body weight gain, which could at least in part be attributed to a reduction of adipose tissue mass. Since the food intake in the HFD+NI group was even higher than in the HFD group, the effect on adiposity cannot be explained by a reduced caloric intake. Analysis of fecal energy content indicated that a comparable amount of energy was excreted, suggesting that the NI may have increased energy expenditure or locomotor activity which should be explored in dedicated future studies.

In humans increased adiposity is associated with elevated leptin and decreased adiponectin plasma concentrations [36], and HFD-treated obese Ldlr.Leiden mice reflected this change in adipokines. Anti-obesogenic dietary or lifestyle regimens can improve adipokine levels [36], and consistent with the anti-obesogenic effects, plasma leptin levels were decreased and adiponectin levels were increased. Previously, we showed that the eHC used herein is a potent nutritional factor that stimulates adiponectin secretion from primary human adipocytes.[13] The observed glucose, insulin and C-peptide lowering effects in mice treated with NI that contains eHC could be in line with observations for other casein hydrolysates [17]. The suggested insulin sensitizing effect may possibly be related to the observed increase in adiponectin. It is well-established that adiponectin also reflect the quality and inflammatory state of fat tissue.[37, 38] It has become obvious that attenuation of chronic inflammation is an important aspect in driving health outcomes such as improving insulin sensitivity, liver function and cardiovascular risk.[39] Consistent with this notion, circulating SAA, a marker of low-grade inflammation [40] SAA was strongly reduced with the NI. SAA is produced and secreted by the liver as well as adipose tissue [41] and SAA directly participates in several processes that contribute to atherosclerosis (e.g. increased oxLDL retention time, stimulation of vascular remodeling).[42, 43] Consistent with the observed reduction of SAA and the reduction in VCAM-1, a vascular marker of inflammation, we found reduced atherosclerotic plaque area in mice that were treated with NI. It is well-established that Ldlr-/- mice develop atherosclerosis after prolonged periods of time, even on a low fat or chow diet.[26] The development of atherosclerosis is driven by LDL cholesterol (which is the primary lipoprotein species in Ldlr-/- mice), and inflammation. Since plasma lipids and the inflammatory state were elevated (typical baseline levels for SAA and VCAM-1 are <10μg/ml and <2μg/ml respectively) in both LFD- and HFD-fed mice, both groups developed atherosclerosis. An important finding of this study is that NI strongly attenuated inflammation in all adipose tissues analyzed. Reduced adipose tissue inflammation may provide a rationale for the attenuated NAFLD and atherosclerosis development since inflammatory factors released from WAT can drive these pathologies.[9, 44] An extensive time-resolved histological analysis of several adipose tissue depots during HFD-induced obesity revealed that inguinal and mesenteric adipose tissue depots of mice are less susceptible to develop HFD induced inflammation when compared to epidydimal WAT because they have a greater ability to expand [9]. Even these less susceptible depots showed a marked reduction of crown like structures upon NI treatment indicating a more generic anti-inflammatory effect of NI that applies to multiple depots. Reduced inflammation of WAT in absence of an effect on its mass was also observed with other anti-inflammatory interventions for NAFLD/NASH such as an inhibitor of caspase-1 [28] or inhibitor of Ccr2.[45]

Our finding that a dietary intervention can attenuate NAFLD development is relevant in light of the rise of obesity and NAFLD as the most common chronic liver disease worldwide which requires preventive (nutrition-based) strategies as an alternative to pharmacotherapy.[46] The beneficial role of extensively casein hydrolysate and LGG has been reported in other studies.[47] For example, the treatment of cow’s milk allergic infants with extensively hydrolyzed casein formula containing LGG resulted in more butyrate-producing bacteria strains.[47] This short-chain fatty acid has been shown to improve intestinal barrier integrity and mucus synthesis [48, 49] which may contribute to observations of attenuated NAFLD and neurodegenerative disorders.[50] With the reported potency of butyrate and producing bacteria, the contribution of butyrate to the hepatoprotective effects of NI merits further investigation.

The explorative follow-up experiment with individual components indicated that the observed hepatoprotective effects of NI cannot be explained by one single constituent, but rather by the combined effects of all constituents, i.e. eHC, LC-PUFAs and LGG. The eHC appeared to exert more pronounced effects when compared to LC-PUFA and LGG at the dose tested, and affected metabolic readouts such as body composition, plasma lipids and insulin. Since the tested NI are constituents of infant formulas, future studies may investigate whether such dietary regimens will prevent obesity and disease development in children, the more so because complications such as NAFLD may already develop in childhood.[51]

## Conclusions

In conclusion, our data provide evidence that a combination of specific NI (extensively hydrolyzed casein, LC-PUFAs ARA and DHA, LGG) can reduce obesity and metabolic diet-induced inflammation at organ level (adipose tissue, liver) with pronounced attenuating effects on NAFLD and atherosclerosis. These results advocate research on dietary approaches for the management of obesity and its comorbidities and warrants further studies in humans.

## Supporting information

S1 Table Compositions of experimental diets. Rodent low fat diet composition with 10 kcal% fat, a high fat diet composition with 46 kcal% fat and an isocaloric high fat diet composition with an extensively hydrolyzed casein and long-chain polyunsaturated fatty acids Docosahexaenoic acid (0.083%) and Arachidonic acid (0.166%).
(TIF)

S1 Fig Representative photomicrographs of cross-sections in the aortic valve area with lumen (L) and representative atherosclerotic lesions. The LFD and HFD groups showed intimal thickening with pronounced atherosclerotic lesions as indicated (black arrows). Intervention with the combination of nutritional ingredients (extensively hydrolyzed casein, long-chain polyunsaturated fatty acids Docosahexaenoic acid, Arachidonic acid and probiotic *Lactobacillus Rhamnosus* GG) attenuated atherosclerosis development with mild intimal thickening and smaller lesions (white arrow).
(TIF)

## Abbreviations

ALAT: Alanine aminotransferase
ARA: Arachidonic acid
CVD: Cardiovascular disease
DHA: Docosahexaenoic acid
eHC: Extensively hydrolyzed casein
HFD: High fat diet
LCPUFA: Long chain polyunsaturated fatty acid
LFD: Low fat diet
LGG: Lactobacillus Rhamnosus *GG*
NAFLD: Non-alcohol fatty liver disease
NI: Nutritional ingredients
SAA: Serum Amyloid A
VCAM: Vascular cell adhesion molecule

## References

[1] Ng M, Fleming T, Robinson M, Thomson B, Graetz N, Margono C, et al Global, regional, and national prevalence of overweight and obesity in children and adults during 1980–2013: a systematic analysis for the Global Burden of Disease Study 2013. Lancet. 2014;384(9945):766–81. Epub 2014/06/02. doi: 10.1016/S0140-6736(14)60460-8 24880830 10.1016/S0140-6736(14)60460-8 PMC4624264

[2] Yates T, Davies MJ, Khunti K. Obesity and chronic disease in younger people: an unfolding crisis. The British journal of general practice: the journal of the Royal College of General Practitioners. 2012;62(594):4–5. Epub 2012/04/24. 22520657 10.3399/bjgp12X616201 PMC3252513

[3] Dietz WH. Critical periods in childhood for the development of obesity. The American journal of clinical nutrition. 1994;59(5):955–9. Epub 1994/05/01. 8172099 10.1093/ajcn/59.5.955

[4] Astrup A, Brand-Miller J. Diet composition and obesity. Lancet. 2012;379(9821):1100; author reply -1. Epub 2012/03/27. 10.1016/S0140-6736(12)60456-5 22444397

[5] Delzenne NM, Neyrinck AM, Backhed F, Cani PD. Targeting gut microbiota in obesity: effects of prebiotics and probiotics. Nature reviews Endocrinology. 2011;7(11):639–46. Epub 2011/08/10. doi: 10.1038/nrendo.2011.126 21826100 10.1038/nrendo.2011.126

[6] Luke A, Cooper RS. Physical activity does not influence obesity risk: time to clarify the public health message. International journal of epidemiology. 2013;42(6):1831–6. Epub 2014/01/15. doi: 10.1093/ije/dyt159 24415616 10.1093/ije/dyt159

[7] Prentice A, Jebb S. Energy intake/physical activity interactions in the homeostasis of body weight regulation. Nutrition reviews. 2004;62(7 Pt 2):S98–104. Epub 2004/09/25. 15387474 10.1111/j.1753-4887.2004.tb00095.x

[8] van der Heijden RA, Sheedfar F, Morrison MC, Hommelberg PP, Kor D, Kloosterhuis NJ, et al High-fat diet induced obesity primes inflammation in adipose tissue prior to liver in C57BL/6j mice. Aging. 2015;7(4):256–68. Epub 2015/05/17. doi: 10.18632/aging.100738 25979814 10.18632/aging.100738 PMC4429090

[9] Mulder P, Morrison MC, Wielinga PY, van Duyvenvoorde W, Kooistra T, Kleemann R. Surgical removal of inflamed epididymal white adipose tissue attenuates the development of non-alcoholic steatohepatitis in obesity. Int J Obes (Lond). 2015. Epub 2015/10/27. 10.1038/ijo.2015.226 PMC4827008 26499443

[10] Romeo GR, Lee J, Shoelson SE. Metabolic syndrome, insulin resistance, and roles of inflammation—mechanisms and therapeutic targets. Arteriosclerosis, thrombosis, and vascular biology. 2012;32(8):1771–6. Epub 2012/07/21. doi: 10.1161/ATVBAHA.111.241869 22815343 10.1161/ATVBAHA.111.241869 PMC4784686

[11] Godfrey KM, Gluckman PD, Hanson MA. Developmental origins of metabolic disease: life course and intergenerational perspectives. Trends in endocrinology and metabolism: TEM. 2010;21(4):199–205. Epub 2010/01/19. doi: 10.1016/j.tem.2009.12.008 20080045 10.1016/j.tem.2009.12.008

[12] von Berg A, Filipiak-Pittroff B, Kramer U, Link E, Bollrath C, Brockow I, et al Preventive effect of hydrolyzed infant formulas persists until age 6 years: long-term results from the German Infant Nutritional Intervention Study (GINI). The Journal of allergy and clinical immunology. 2008;121(6):1442–7. Epub 2008/06/10. doi: 10.1016/j.jaci.2008.04.021 18539195 10.1016/j.jaci.2008.04.021

[13] Berni Canani R, Nocerino R, Terrin G, Coruzzo A, Cosenza L, Leone L, et al Effect of Lactobacillus GG on tolerance acquisition in infants with cow's milk allergy: a randomized trial. The Journal of allergy and clinical immunology. 2012;129(2):580–2, 2 e1–5. Epub 2011/11/15. doi: 10.1016/j.jaci.2011.10.004 22078573 10.1016/j.jaci.2011.10.004

[14] Lapillonne A, Pastor N, Zhuang W, Scalabrin DM. Infants fed formula with added long chain polyunsaturated fatty acids have reduced incidence of respiratory illnesses and diarrhea during the first year of life. BMC pediatrics. 2014;14:168 Epub 2014/07/06. doi: 10.1186/1471-2431-14-168 24989353 10.1186/1471-2431-14-168 PMC4098921

[15] Drover J, Hoffman DR, Castaneda YS, Morale SE, Birch EE. Three randomized controlled trials of early long-chain polyunsaturated Fatty Acid supplementation on means-end problem solving in 9-month-olds. Child development. 2009;80(5):1376–84. Epub 2009/09/22. doi: 10.1111/j.1467-8624.2009.01339.x 19765006 10.1111/j.1467-8624.2009.01339.x PMC2757317

[16] Clausen MR, Zhang X, Yde CC, Ditlev DB, Lillefosse HH, Madsen L, et al Intake of hydrolyzed casein is associated with reduced body fat accretion and enhanced phase II metabolism in obesity prone C57BL/6J mice. PloS one. 2015;10(3):e0118895 Epub 2015/03/05. doi: 10.1371/journal.pone.0118895 25738501 10.1371/journal.pone.0118895 PMC4349863

[17] Lillefosse HH, Tastesen HS, Du ZY, Ditlev DB, Thorsen FA, Madsen L, et al Hydrolyzed casein reduces diet-induced obesity in male C57BL/6J mice. The Journal of nutrition. 2013;143(9):1367–75. Epub 2013/07/12. doi: 10.3945/jn.112.170415 23843475 10.3945/jn.112.170415

[18] Lambers TT, Gloerich J, van Hoffen E, Alkema W, Hondmann DH, van Tol EA. Clustering analyses in peptidomics revealed that peptide profiles of infant formulae are descriptive. Food science & nutrition. 2015;3(1):81–90. Epub 2015/02/05. 25648153 10.1002/fsn3.196 PMC4304566

[19] Flachs P, Rossmeisl M, Bryhn M, Kopecky J. Cellular and molecular effects of n-3 polyunsaturated fatty acids on adipose tissue biology and metabolism. Clin Sci (Lond). 2009;116(1):1–16. Epub 2008/11/29. 19037880 10.1042/CS20070456

[20] Gray B, Steyn F, Davies PS, Vitetta L. Omega-3 fatty acids: a review of the effects on adiponectin and leptin and potential implications for obesity management. European journal of clinical nutrition. 2013;67(12):1234–42. Epub 2013/10/17. doi: 10.1038/ejcn.2013.197 24129365 10.1038/ejcn.2013.197

[21] Lopez-Alarcon M, Martinez-Coronado A, Velarde-Castro O, Rendon-Macias E, Fernandez J. Supplementation of n3 long-chain polyunsaturated fatty acid synergistically decreases insulin resistance with weight loss of obese prepubertal and pubertal children. Archives of medical research. 2011;42(6):502–8. Epub 2011/12/06. doi: 10.1016/j.arcmed.2011.06.010 22136960 10.1016/j.arcmed.2011.06.010

[22] Juarez-Hernandez E, Chavez-Tapia NC, Uribe M, Barbero-Becerra VJ. Role of bioactive fatty acids in nonalcoholic fatty liver disease. Nutrition journal. 2016;15(1):72 Epub 2016/08/04. doi: 10.1186/s12937-016-0191-8 27485440 10.1186/s12937-016-0191-8 PMC4970250

[23] Wielinga PY, Harthoorn LF, Verschuren L, Schoemaker MH, Jouni ZE, van Tol EA, et al Arachidonic acid/docosahexaenoic acid-supplemented diet in early life reduces body weight gain, plasma lipids, and adiposity in later life in ApoE*3Leiden mice. Molecular nutrition & food research. 2012;56(7):1081–9. Epub 2012/05/23. 22611002 10.1002/mnfr.201100762

[24] Hojsak I, Snovak N, Abdovic S, Szajewska H, Misak Z, Kolacek S. Lactobacillus GG in the prevention of gastrointestinal and respiratory tract infections in children who attend day care centers: a randomized, double-blind, placebo-controlled trial. Clin Nutr. 2010;29(3):312–6. Epub 2009/11/10. doi: 10.1016/j.clnu.2009.09.008 19896252 10.1016/j.clnu.2009.09.008

[25] Ritze Y, Bardos G, Claus A, Ehrmann V, Bergheim I, Schwiertz A, et al Lactobacillus rhamnosus GG protects against non-alcoholic fatty liver disease in mice. PloS one. 2014;9(1):e80169 Epub 2014/01/30. doi: 10.1371/journal.pone.0080169 24475018 10.1371/journal.pone.0080169 PMC3903470

[26] Verschuren L, Kooistra T, Bernhagen J, Voshol PJ, Ouwens DM, van Erk M, et al MIF deficiency reduces chronic inflammation in white adipose tissue and impairs the development of insulin resistance, glucose intolerance, and associated atherosclerotic disease. Circulation research. 2009;105(1):99–107. Epub 2009/05/30. doi: 10.1161/CIRCRESAHA.109.199166 19478200 10.1161/CIRCRESAHA.109.199166 PMC2717797

[27] Liang W, Menke AL, Driessen A, Koek GH, Lindeman JH, Stoop R, et al Establishment of a general NAFLD scoring system for rodent models and comparison to human liver pathology. PloS one. 2014;9(12):e115922 Epub 2014/12/24. doi: 10.1371/journal.pone.0115922 25535951 10.1371/journal.pone.0115922 PMC4275274

[28] Morrison MC, Mulder P, Salic K, Verheij J, Liang W, van Duyvenvoorde W, et al Intervention with a caspase-1 inhibitor reduces obesity-associated hyperinsulinemia, non-alcoholic steatohepatitis and hepatic fibrosis in LDLR-/-.Leiden mice. Int J Obes (Lond). 2016;40(9):1416–23. Epub 2016/04/29. 27121255 10.1038/ijo.2016.74 PMC5022108

[29] Bligh EG D W. A rapid method of total lipid extraction and purification. Can J Biochem Physiol. 1959;37(8):911–7. doi: 10.1139/o59-099 13671378 10.1139/o59-099

[30] Lowry OH R N, Farr AL, Randall RJ. Protein measurement with the Folin phenol reagent. The Journal of biological chemistry. 1951;193(1):265–75. 14907713

[31] Morrison MC, Liang W, Mulder P, Verschuren L, Pieterman E, Toet K, et al Mirtoselect, an anthocyanin-rich bilberry extract, attenuates non-alcoholic steatohepatitis and associated fibrosis in ApoE (*)3Leiden mice. Journal of hepatology. 2015;62(5):1180–6. Epub 2014/12/17. doi: 10.1016/j.jhep.2014.12.011 25514555 10.1016/j.jhep.2014.12.011

[32] Kooistra T, Verschuren L, de Vries-van der Weij J, Koenig W, Toet K, Princen HM, et al Fenofibrate reduces atherogenesis in ApoE*3Leiden mice: evidence for multiple antiatherogenic effects besides lowering plasma cholesterol. Arteriosclerosis, thrombosis, and vascular biology. 2006;26(10):2322–30. Epub 2006/07/29. doi: 10.1161/01.ATV.0000238348.05028.14 16873727 10.1161/01.ATV.0000238348.05028.14

[33] Hu FB, Manson JE, Willett WC. Types of dietary fat and risk of coronary heart disease: a critical review. Journal of the American College of Nutrition. 2001;20(1):5–19. Epub 2001/04/11. 11293467 10.1080/07315724.2001.10719008

[34] Radonjic M, Wielinga PY, Wopereis S, Kelder T, Goelela VS, Verschuren L, et al Differential effects of drug interventions and dietary lifestyle in developing type 2 diabetes and complications: a systems biology analysis in LDLr-/- mice. PloS one. 2013;8(2):e56122 Epub 2013/03/05. doi: 10.1371/journal.pone.0056122 23457508 10.1371/journal.pone.0056122 PMC3574110

[35] Zadelaar S, Kleemann R, Verschuren L, de Vries-Van der Weij J, van der Hoorn J, Princen HM, et al Mouse models for atherosclerosis and pharmaceutical modifiers. Arteriosclerosis, thrombosis, and vascular biology. 2007;27(8):1706–21. Epub 2007/06/02. doi: 10.1161/ATVBAHA.107.142570 17541027 10.1161/ATVBAHA.107.142570

[36] Calder PC, Ahluwalia N, Albers R, Bosco N, Bourdet-Sicard R, Haller D, et al A consideration of biomarkers to be used for evaluation of inflammation in human nutritional studies. The British journal of nutrition. 2013;109 Suppl 1:S1–34. Epub 2013/02/01. 10.1017/S0007114512005119 23343744

[37] Romacho T, Elsen M, Rohrborn D, Eckel J. Adipose tissue and its role in organ crosstalk. Acta Physiol (Oxf). 2014;210(4):733–53. Epub 2014/02/06. 24495317 10.1111/apha.12246

[38] Mulder P, Morrison MC, Verschuren L, Liang W, van Bockel JH, Kooistra T, et al Reduction of obesity-associated white adipose tissue inflammation by rosiglitazone is associated with reduced non-alcoholic fatty liver disease in LDLr-deficient mice. Scientific reports. 2016;6:31542 Epub 2016/08/23. doi: 10.1038/srep31542 27545964 10.1038/srep31542 PMC4992869

[39] Monteiro R, Azevedo I. Chronic inflammation in obesity and the metabolic syndrome. Mediators of inflammation. 2010;2010. Epub 2010/08/14. 10.1155/2010/289645 PMC2913796 20706689

[40] Poitou C, Coussieu C, Rouault C, Coupaye M, Cancello R, Bedel JF, et al Serum amyloid A: a marker of adiposity-induced low-grade inflammation but not of metabolic status. Obesity (Silver Spring). 2006;14(2):309–18. Epub 2006/03/31. 16571858 10.1038/oby.2006.40

[41] Kleemann R, Verschuren L, van Erk MJ, Nikolsky Y, Cnubben NH, Verheij ER, et al Atherosclerosis and liver inflammation induced by increased dietary cholesterol intake: a combined transcriptomics and metabolomics analysis. Genome biology. 2007;8(9):R200 Epub 2007/09/26. doi: 10.1186/gb-2007-8-9-r200 17892536 10.1186/gb-2007-8-9-r200 PMC2375038

[42] Krishack PA, Bhanvadia CV, Lukens J, Sontag TJ, De Beer MC, Getz GS, et al Serum Amyloid A Facilitates Early Lesion Development in Ldlr-/- Mice. Journal of the American Heart Association. 2015;4(7). Epub 2015/07/19. 10.1161/JAHA.115.001858 PMC4608070 26187995

[43] Fuijkschot WW, Morrison MC, van der Linden R, Krijnen PA, Zethof IP, Theyse LF, et al Orthopedic surgery increases atherosclerotic lesions and necrotic core area in ApoE-/- mice. Atherosclerosis. 2016;255:164–70. Epub 2016/11/09. doi: 10.1016/j.atherosclerosis.2016.07.909 27825629 10.1016/j.atherosclerosis.2016.07.909

[44] Rajala MW S P. Minireview: The adipocyte—at the crossroads of energy homeostasis, inflammation, and atherosclerosis. Endocrinology. 2003;144(9):3765–73. doi: 10.1210/en.2003-0580 12933646 10.1210/en.2003-0580

[45] Mulder P, van den Hoek AM, Kleemann R. The CCR2 Inhibitor Propagermanium Attenuates Diet-Induced Insulin Resistance, Adipose Tissue Inflammation and Non-Alcoholic Steatohepatitis. PloS one. 2017;12(1):e0169740 Epub 2017/01/12. doi: 10.1371/journal.pone.0169740 28076416 10.1371/journal.pone.0169740 PMC5226841

[46] Brumbaugh DE, Friedman JE. Developmental origins of nonalcoholic fatty liver disease. Pediatric research. 2014;75(1–2):140–7. Epub 2013/11/07. doi: 10.1038/pr.2013.193 24192698 10.1038/pr.2013.193 PMC4081536

[47] Berni Canani R, Sangwan N, Stefka AT, Nocerino R, Paparo L, Aitoro R, et al Lactobacillus rhamnosus GG-supplemented formula expands butyrate-producing bacterial strains in food allergic infants. The ISME journal. 2015. Epub 2015/09/24. 10.1038/ismej.2015.151 PMC4817673 26394008

[48] Thibault R, Blachier F, Darcy-Vrillon B, de Coppet P, Bourreille A, Segain JP. Butyrate utilization by the colonic mucosa in inflammatory bowel diseases: a transport deficiency. Inflammatory bowel diseases. 2010;16(4):684–95. Epub 2009/09/24. doi: 10.1002/ibd.21108 19774643 10.1002/ibd.21108

[49] Canfora EE, Jocken JW, Blaak EE. Short-chain fatty acids in control of body weight and insulin sensitivity. Nature reviews Endocrinology. 2015;11(10):577–91. Epub 2015/08/12. doi: 10.1038/nrendo.2015.128 26260141 10.1038/nrendo.2015.128

[50] Arnoldussen IA, Wiesmann M, Pelgrim CE, Wielemaker EM, van Duyvenvoorde W, Amaral-Santos PL, et al Butyrate restores HFD-induced adaptations in brain function and metabolism in mid-adult obese mice. Int J Obes (Lond). 2017. Epub 2017/02/22. 10.1038/ijo.2017.52 28220041

[51] Nobili V, Svegliati-Baroni G, Alisi A, Miele L, Valenti L, Vajro P. A 360-degree overview of paediatric NAFLD: recent insights. Journal of hepatology. 2013;58(6):1218–29. Epub 2012/12/15. doi: 10.1016/j.jhep.2012.12.003 23238106 10.1016/j.jhep.2012.12.003

