# Supplementary figures and images for: A casein hydrolysate based formulation attenuates obesity and associated non-alcoholic fatty liver disease and atherosclerosis in LDLr-/-.Leiden mice
Source: PLoS One. 2017 Jul 5;12(7):e0180648. doi: 10.1371/journal.pone.0180648 (PMC5498059; doi:10.1371/journal.pone.0180648)

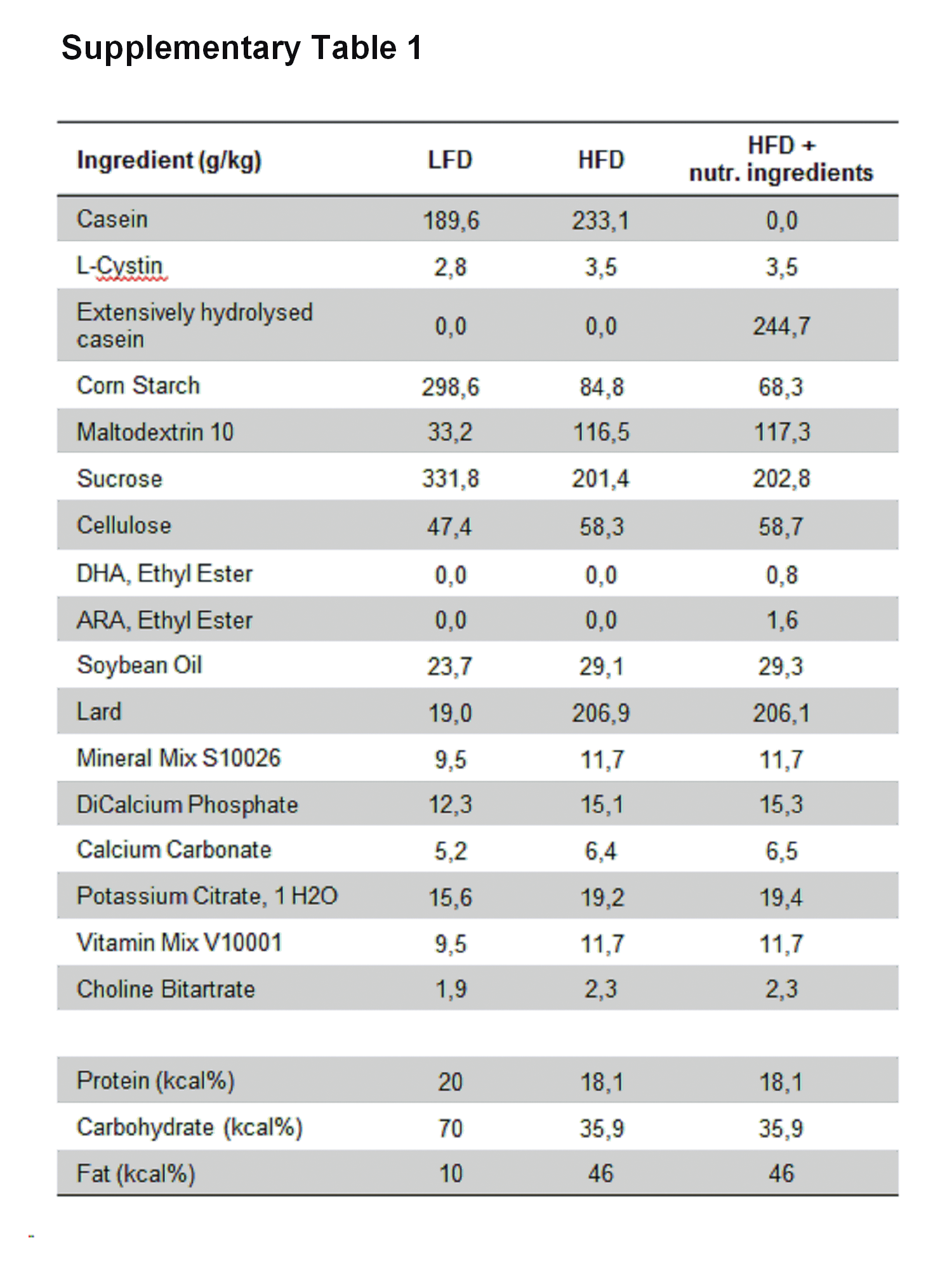

Supplement: S1 Table — Rodent low fat diet composition with 10 kcal% fat, a high fat diet composition with 46 kcal% fat and an isocaloric high fat diet composition with an extensively hydrolyzed casein and long-chain polyunsaturated fatty acids Docosahexaenoic acid (0.083%) and Arachidonic acid (0.166%). (TIF) [file pone.0180648.s001.tif]

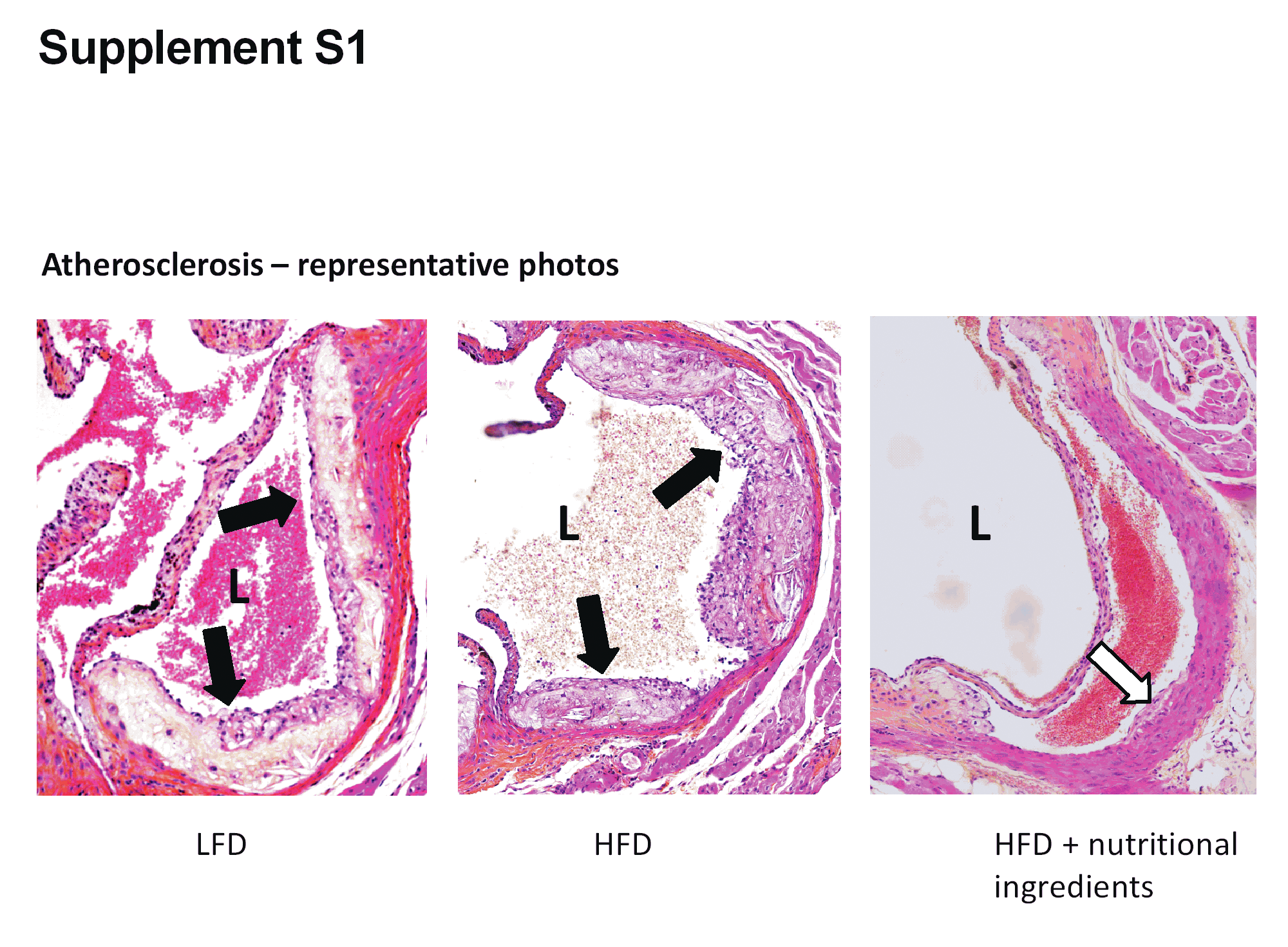

Supplement: S1 Fig — The LFD and HFD groups showed intimal thickening with pronounced atherosclerotic lesions as indicated (black arrows). Intervention with the combination of nutritional ingredients (extensively hydrolyzed casein, long-chain polyunsaturated fatty acids Docosahexaenoic acid, Arachidonic acid and probiotic Lactobacillus Rhamnosus GG) attenuated atherosclerosis development with mild intimal thickening and smaller lesions (white arrow). (TIF) [file pone.0180648.s002.tif]
